# Development and psychometric properties of a self-medication behavior inventory

**DOI:** 10.3389/fpsyg.2024.1366284

**Published:** 2024-05-06

**Authors:** Julio C. Penagos-Corzo, Melissa J. Ortiz-Barrero, Reneé Hernández-Ramírez, Yavne Ochoa-Ramírez, Regina González Ehlinger, Andrés M. Pérez-Acosta

**Affiliations:** ^1^Department of Psychology, Universidad de las Américas Puebla, San Andrés Cholula, Mexico; ^2^Universidad Nacional Abierta y a Distancia, Bogotá, Colombia; ^3^Observatory of Self-medication Behavior, Psychology Programme, School of Medicine and Health Sciences, Universidad del Rosario, Bogotá, Colombia

**Keywords:** self-medication, avoidance, prevention, psychometric validation, attitudes, social influence

## Abstract

**Introduction:**

Self-medication is a prevalent behavior with significant health implications. Understanding its psychosocial determinants can inform preventative strategies and interventions.

**Methods:**

We evaluated the psychometric properties of the Self-Medication Behavior Inventory (SMBI-9) in a binational study with 779 Colombian and Mexican participants. Concurrent validity was assessed through correlations with related inventories, and confirmatory factor analysis tested the proposed four-factor model.

**Results:**

The SMBI-9 demonstrated high model fit (CFI = 0.995, TLI = 0.991) and invariance across countries. The factors-Social Influence, Attitude toward Medicine, Avoidance, and Prevention-varied significantly with knowledge of medicine, schooling, health insurance status and gender, underscoring the role of social and personal beliefs in self-medication practices.

**Discussion:**

SMBI-9 emerged as a reliable tool for capturing the multifaceted nature of self-medication behaviors. Findings highlight the influence of social norms and personal attitudes, suggesting targeted approaches for behavioral interventions.

## Graphical Abstract

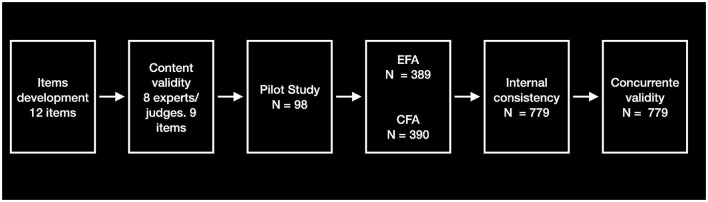

## 1 Introduction

The present study addresses self-medication as the use of medications in the absence of a medical prescription. Such behavior can be positive and related to self-care (Ruiz-Sternberg and Pérez-Acosta, 2011; Baracaldo-Santamaría et al., 2022; Bertsche et al., 2023). In fact, it has been reported to be a behavior present in various species - from arthropods to humans - that serves to combat contextual threats, such as diseases and their accompanying symptoms (Huffman, 2003; Gasco et al., 2016). Even findings of genetic predisposition for increased self-medication have been reported (Lerman et al., 1998). However, it also has significant health risks (Camargo Rubio, 2023). Among these are bacterial resistance and inadequate diagnostics (Zambrano Barriga and Cusme Torres, 2023), lack of knowledge of adverse reactions and dangerous interactions (Ekor, 2014), as well as drug dependence or abuse (Ruiz, 2010; Pokida and Zybunovskaya, 2023). In addition to the risks, there is a high prevalence of self-medication (Alves et al., 2021). It has been estimated, for example, that during the COVID-19 pandemic, self-medication had an overall prevalence of 48% (Kazemioula et al., 2022). Even self-medication of antibiotics reaches levels of almost 55% in countries such as Peru (Benites-Meza et al., 2023) or 50% in Indonesia (Karuniawati et al., 2023). Globally, it varies according to the economic development of the country, but can reach levels of more than 80% in some middle-income regions (Ahmed et al., 2023).

Due to the existing risks in the behavior of self-medication and its prevalence, it is relevant to identify the variables that contribute to explaining the behavior of self-medication. In this sense, it has been suggested that both pain avoidance (Zambrano Barriga and Cusme Torres, 2023) and better performance in some capacity (Rabiner et al., 2009) are some of the causes of self-medication. In the same direction, there are reports that indicate that workers may adopt risky behaviors in self-medication in order to avoid pain or increase their productivity (Castillo Martínez and Pérez-Acosta, 2021). Outside the work or productive environment, long-term ailments and physical pain are predictors of self-medication, mainly in older adults (Brandão et al., 2020). On the other hand, in young populations, minor pain is associated with frequent use of self-medicated analgesics (Ibrahim et al., 2014). In fact, it has been reported that analgesics are the most commonly used drugs in self-medication (James et al., 2006). Other variables, in addition to avoidance and prevention, may be involved in self-medication. For example, attitudes toward medical practice and knowledge of a drug's effects (Grigoryan et al., 2007). It is also possible that direct drug advertising plays a role. Although it has been reported to have a limited impact on consumer choices in the case of antidepressants, it is also possible that direct advertising of drugs plays a role (Donohue and Berndt, 2004), this is not the case for drugs that do not require a prescription to be purchased and are highly advertised (Burak and Damico, 2000). From the aforementioned, attitudes, media influence, as well as avoidance and prevention are involved in self-medication behavior. The above can be supported by the Health Belief Model (Becker, 1974; Rosenstock, 2005) and the theory of planned behavior (Ajzen, 1991). From the perspective of the Health Belief Model, people will be willing to take preventive actions for health if they have a perception of high risk of getting sick, and the benefits of taking such actions outweigh the costs (Etheridge et al., 2023). Furthermore, from the theory of action reasoning, it is postulated that behavioral intentions are influenced by attitudes toward behavior, subjective norms, and perceived control over behavior (Ajzen, 1991). Attitudes toward self-medication may include beliefs about the efficacy and safety of self-medication without a prescription, as well as assessments of the associated risks and benefits (Hagger et al., 2018). In the case of subjective norms, if a person believes that people significant to them approve of his behavior, in this case self-medication, he is more likely to engage in it (Rivis and Sheeran, 2003). Meanwhile, perceived control is related to the perception of the ease or difficulty of self-medication, which could be influenced by access to medication and the apparent knowledge about its use (Khan et al., 2014).

### 1.1 Avoidance and prevention

In this sense, pain avoidance is a cause of self-medication (Castillo Martínez and Pérez-Acosta, 2021; Zambrano Barriga and Cusme Torres, 2023). Avoidance operate as a form of aversive behavioral control, where the individual emits a response that avoids the aversive stimulus. Such behaviors tend to increase and are further reinforced by the safety signals accompanying avoidance responses, offering positive reinforcement (Domjan and Grau, 2015). In the same direction, there are prevention measures derived from the fear of contracting a contagious disease (Zheng et al., 2023). Furthermore, individuals might seek to avoid the discomfort or inconvenience associated with long waits at health clinic (Sharif et al., 2012) or the avoidance of aversive states of negative affect (Kassel, 2010).

### 1.2 Influence

The selection of a product can be significantly swayed by advertising strategies, particularly those targeting social norms (Melnyk et al., 2019). Medications are not immune to such influence, as marketing affects the practice of self-medication (Fuentes Albarrán and Villa Zapata, 2008). Advertisements, in fact, play a role in the self-prescription of a drug (Burak and Damico, 2000). This dynamic could partially explain why one-third of drug sales revenue is devoted to marketing (Koinig et al., 2017). In addition, self-medication practices are shaped by recommendations encountered on social media platforms (Zeb et al., 2022), as well as recommendations from people close to them, which may carry greater weight than information from a healthcare professional (Anghel and Craciun, 2013).

### 1.3 Attitude

The evidence regarding the impact of attitudes toward self-medication on the practice itself is mixed (Sulistyowatia et al., 2022). This inconsistency might stem from the fact that attitudes do not always predict behavior (Myers and Twenge, 2021), as the relationship may be inverse: attitudes arise from behaviors for which people feel responsible (Harmon-Jones and Harmon-Jones, 2019). In other words, behavior precedes attitude. However, it is also plausible that it is attitudes toward medicine, medical services, or even medications that are related to self-medication behavior. For example, fear of adverse drug effects has been reported to be a cause of self-medication (Parihar et al., 2018). This fear is also related to adherence (Krueger et al., 2005), which is linked to self-medication practices (Mir, 2018). Additionally, if the person has had negative experiences with health care providers, individuals may resort to self-medication as an alternative (Dassah et al., 2018).

As noted above, self-medication behavior, despite its potential benefits, has highly relevant risks (Hughes et al., 2001; Kretchy et al., 2021; Chiniard et al., 2023) and a high prevalence (Alhomoud et al., 2017; Alomaim et al., 2023). Despite this, explanations for such behavior are insufficient, as empirical approaches to its assessment are scarce. For example, in August 2023, the APA PsycTest database, which has more than 70,000 records of scientific reports on psychometric instruments, had only four records of measures related to self-medication. Three of them were related to substance use, and one related to the ability to provide oneself with medication prescribed by a physician. It is possible to find questionnaires related to self-medication in other indexes or databases, but in general, they are descriptive, indirect, or lack evaluation of their psychometric properties. Although one could be located, it only evaluates the adolescent population (Ortega Latorre et al., 2018). Therefore, due to the risks of self-medication behavior and the absence of instruments that assess such behavior, and also help to explain it, the purpose of this study is to design and evaluate the validity and reliability of an inventory on self-medication behavior.

## 2 Materials and methods

This research was conducted using a quantitative, non-experimental methodology, framed within a cross-sectional design. The psychometric study process was delineated into six detailed phases to ensure the thoroughness and rigor of the analysis. The first phase focused on the development of the items. The second phase involved an evaluation of content validity by a panel of expert judges, who examined each item to confirm its relevance and appropriateness. The third phase consisted of a preliminary evaluation through a pilot study, aimed at testing the effectiveness and clarity of the developed items. Subsequently, in the fourth phase, factorial analyses were conducted to explore the underlying structure of the data and thus determine and confirm the retained factors. The fifth phase was dedicated to the analysis of internal consistency of the identified factors and the entire instrument. Finally, the sixth and last phase addressed the analysis of concurrent validity, linking the instrument's results with established external measures to confirm its empirical validity. Data collection for the current study was conducted between October 2022 and January 2023. The graphical diagram of the study phases is shown in the Graphical Abstract.

### 2.1 Participants

A total of 779 participants were selected by availability, comprising 267 Mexicans and 512 Colombians. The gender distribution included 639 women, 135 men, three individuals who described their gender as “other,” and two who preferred not to respond. The average age was 29.1 years (standard deviation = 11.7) for women and 31.8 years (standard deviation = 13.83) for men. Participants were recruited using two main methods: (a) Professors from universities in Mexico and Colombia invited their students to complete the questionnaires by sharing the link during their courses. (b) Additionally, the questionnaire link was disseminated through the official social media channels of the Psychology department at the university. Moreover, ~100 participants were specifically recruited through the Prolific platform, where the geographical areas of interest and the age of the participants were defined to align with the study's requirements.

The sample was randomly divided into two subsamples. One (SsE) for exploratory factor analysis (*N* = 389) and the other (SsC) for confirmatory factor analysis (*N* = 390).

Other demographic characteristics of the sample are described in Table 1, both for SsE, SsC, and total.

**Table 1:** Characteristics of the subsamples and the complete sample.

**Sample**	**SsE**		**SsC**		**Total**	
Overall		*N =* 390		*N =* 389		*N =* 779
Education	Elementary/secondary school:	*N =* 2 | 0.5%	Elementary/secondary school:	*N =* 4 | 1%	Elementary/secondary school:	*N =* 6 | 1%
	Completed high school:	*N =* 52 | 13%	Completed high school:	*N =* 42 | 11%	Completed high school:	*N =* 94 | 12%
	Undergraduate students	*N =* 244 | 62.5%	Undergraduate students	*N =* 269 | 69%	Undergraduate students	*N =* 513 | 66%
	Completed bachelor's degree	*N =* 64 | 17%	Completed bachelor's degree	*N =* 49 | 13%	Completed bachelor's degree	*N =* 113 | 15%
	Completed graduate studies	*N =* 28 | 7 %	Completed graduate studies	*N =* 25 | 6%	Completed graduate studies	N= 53 | 6%
Formal knowledge of medicine	No	*N =* 357 | 92%	No	*N =* 357 | 92%	No	*N =* 714 | 92%
Yes	*N =* 33 | 8%	Yes	*N =* 32 | 8%	Yes	*N =* 65 | 8%
Health insurance either public or private	No	*N =* 27 | 7%	No	*N =* 37 | 10%	No	*N =* 64 | 8%
Yes	*N =* 363 | 93%	Yes	*N =* 352 | 90%	Yes	*N =* 715, | 92%
Medical condition or illness that had lasted at least six months	No	*N =* 292 | 75%	No	*N =* 304 | 78%	No	*N =* 596, | 77%
Yes	*N =* 292 | 75%	Yes	*N =* 85 | 22%	Yes	*N =* 183, | 23%

*SsE, Subsample for exploratory factor analysis; SsC, Subsample for confirmatory factor analysis*.

### 2.2 Instruments

*Self-medication behavior inventory (SMBI-9)*, developed in the present study. It consists of nine items and is answered on a seven-point Likert scale, from never to always, where Neve*r* = 0 and Always = 6.

*General self-efficacy scale (GSE)* (Schwarzer et al., 1997). This scale has a Cronbach's alpha = 0.87 and consists of 10 items that are evaluated on a 4-point Likert scale, where 1 = incorrect and 4 = Correct.

*Self-medicating scale (SMS)* (James and French, 2008). This nine-item test is scored on a scale of 1 to 7, where 1 = not severe, and 7 = very severe, with an average Cronbach's alpha of 0.77.

*Perceived social influence on health behavior instrument (PSI-HB)* (Holt et al., 2010). It has an overall alpha of 0.90. It is composed of 10 items, with a 4-point Likert-type response format: strongly disagree, disagree, agree, agree, strongly agree.

*Drug Attitude Inventory (DAI)*. The 10-item Spanish version was used (Robles García et al., 2004). The response options are false and true, which are scored as +1 or −1, depending on the direction of the item. The reported alpha of the version used is 0.57.

### 2.3 Procedure

#### 2.3.1 Item development

The items were generated based on the recommendations of Boateng et al. (2018). For this purpose, a logical or deductive partitioning method was used, which included literature review and evaluation of the indicators of the construct to be measured. The literature review highlighted four dimensions critical to self-medication behavior: Avoidance (Sharif et al., 2012; Castillo Martínez and Pérez-Acosta, 2021; Zambrano Barriga and Cusme Torres, 2023), Prevention (Zheng et al., 2023), Influence (Burak and Damico, 2000; Anghel and Craciun, 2013; Zeb et al., 2022) and Attitude (Krueger et al., 2005; Parihar et al., 2018; Sulistyowatia et al., 2022).

The dimension of Avoidance, influenced by the work of Zambrano Barriga and Cusme Torres (2023), reflects the tendency to evade discomfort or pain, a noted motivator for self-medication. This dimension is rooted in the principle of aversive behavioral control, where behaviors are aimed at avoiding aversive stimuli, with reinforcement coming from accompanying safety signals (Domjan and Grau, 2015).

Prevention, as discussed by Zheng et al. (2023), encompasses actions taken to prevent disease or discomfort (Sharif et al., 2012).

The Influence dimension acknowledges the impact of advertising, social media, and peer recommendations on self-medication practices, indicating the significant role of social norms and marketing (Burak and Damico, 2000; Zeb et al., 2022).

The Attitude dimension incorporates beliefs about the efficacy and safety of self-medication and personal assessments of its risks and benefits. This dimension is informed by studies that link attitudes toward medicine and the healthcare system to self-medication practices (Krueger et al., 2005; Sulistyowatia et al., 2022).

Based on these dimensions, 12 items were created, three per dimension, and then evaluated by experts to assess their content validity. This process aimed to ensure that the items accurately represent the construct of self-medication behavior as informed by the literature.

#### 2.3.2 Content validity

To assess content validity, eight experts were called upon to act as judges. These experts were academics with lines of research closely linked to the object of study. The experts were asked to rate the relevance of each item based on a three-level criterion (Lawshe, 1975) (a) the item is essential, (b) the item is useful but not essential, and (c) the item is not relevant. The content validity coefficient was calculated using the Tristán-López (2008) algorithm. As a result of this process, three items were eliminated, leaving a total of nine items.

#### 2.3.3 Pilot study

The nine items were tested in a pilot sample of 98 participants selected through an invitation made in social media of the psychology department of the university of affiliation of one of the researchers and in professional social networks.

#### 2.3.4 Application of the instruments

Following this analysis, the SMBI-9 was administered online, along with the other instruments, to the sample of 779 participants. The other instruments were selected to assess concurrent validity. Positive relationships were hypothesized between the SMBI-9 with the GSE, the PSI-HB, and the DAI, and negative relationships between the SMS and the SMBI-9.

#### 2.3.5 Statistical analysis

Statistical analyses were performed in SPSS v 28 (SPSS Inc, 2021), AMOS v 29 (Arbuckle, 2022) and Jamovi v 2.3.21 (The Jamovi Project, 2023). For the exploratory factor analysis, principal axis factorization with PROMAX rotation was used, considering that the factors were correlated. In the factor extraction process, the main criteria were: eigenvalue of the parallel analysis higher than the simulated random values (Horn, 1965) and factor loadings >0.40. The confirmatory factor analysis was evaluated using the comparative fit index (CFI), the Tucker-Lewis index (TCI) and the root mean square error of approximation (RMSEA). Regarding the CFI and TLI indices, there is a general consensus to use a cutoff point of 0.95 as an indicator of optimal fit, and values below 0.06 for the RMSEA (Hu and Bentler, 1999; Barrett, 2007). Internal consistency was assessed through the McDonald omega. The omega coefficient was preferred since, unlike the alpha coefficient, it is not affected by the number of items, is compatible with factor loadings and is considered superior to the alpha coefficient (Trizano-Hermosilla and Alvarado, 2016; Deng and Chan, 2017).

## 3 Results

### 3.1 Pilot study findings

A total of 67 women with a mean age of 28.87 (SD = 15.35) and 29 men with a mean age of 32.86 (15.08), made up this sample. Two people chose the option “Other” in gender, and their ages were 19 and 21 years. Of the females, 55.07% were undergraduate students, 30.43% were undergraduate graduates, 7.25% had graduate degrees, and an equal percentage had only a bachelor's degree. A total of 51.72% were bachelor's degree graduates, 24.14% were undergraduate students, 17.24% were graduate students, and 6.9% had a bachelor's degree as their highest level of education. The sample was divided into two groups to test the discrimination capacity of the items: One with 27% of the participants who obtained the highest scores and another with 27% of the participants who obtained the lowest scores. With these two groups, item discrimination ability was analyzed through a t-test, comparing the high vs. low group on each item (Penagos-Corzo et al., 2019). All items showed differences <0.01. Derived from the analysis of the pilot study and interviews with study participants, it was decided to move from a five-point Likert scale to a seven-point Likert scale. This would allow greater variability and more precision, since the response options at the extremes of the scale were “Never or almost never” and “Always or almost always.” These response options were split into two. In the final version of the instrument, the extreme was left as “Never”, followed by the option “Almost never.” The same at the other end, where the option was “Always”, preceded by the option “Almost always.”

### 3.2 Exploratory factor analysis

**Figure 1:**
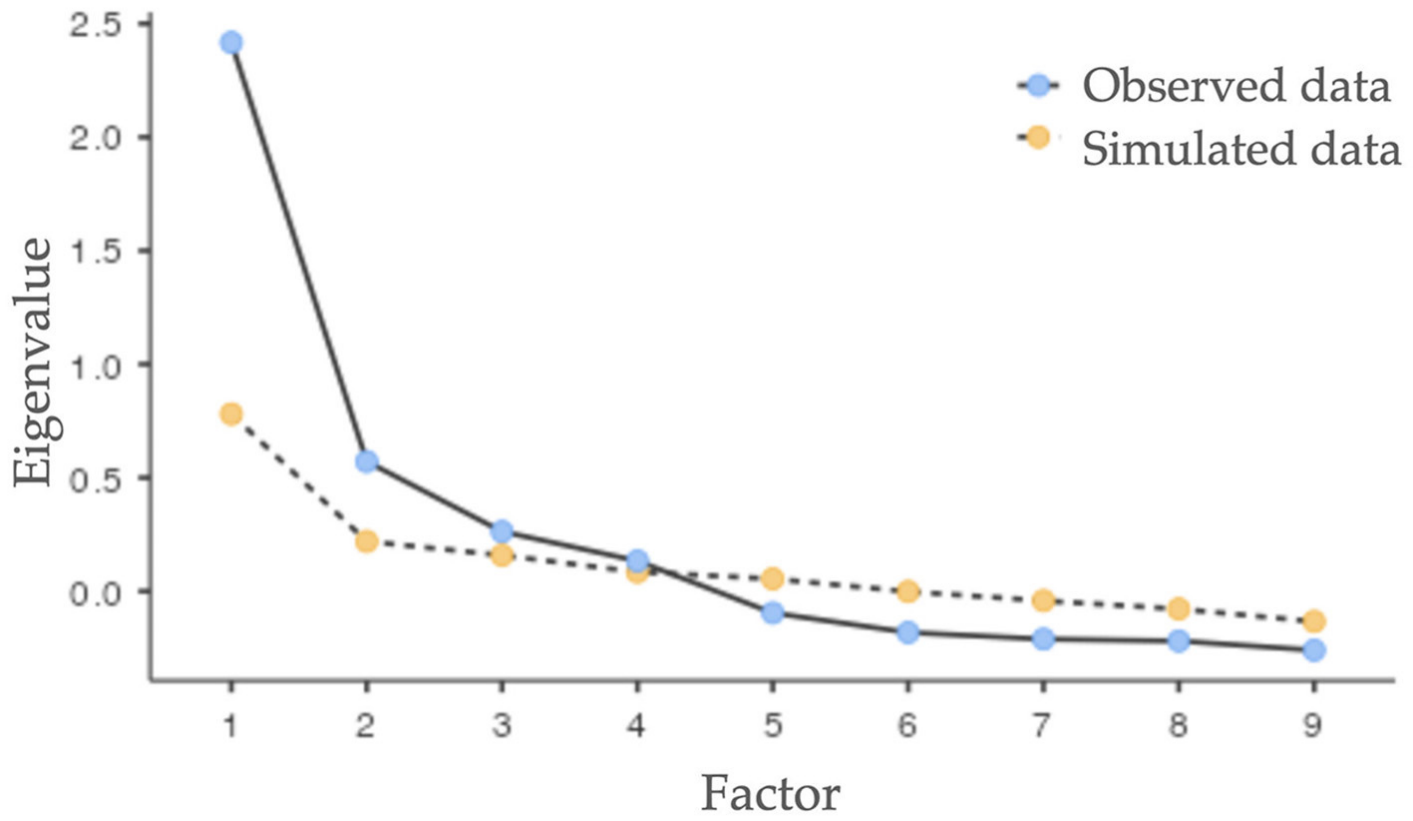
Parallel analysis scree plot from exploratory factor analysis for the Self-Medication Behavior Inventory (SMBI-9).

Prior to the exploratory factor analysis (EFA) performed with the SsE sample, the homogeneity of the items in this sample was tested. The data indicate an average of 0.442 for the corrected item-total correlation, with a range between 0.343 and 0.546. The EFA indicated a KMO of 0.786, and Bartlett's test of sphericity was significant [*X*^2^(36) = 720, *p* < 0.001], indicating that it was pertinent to perform the factor analysis. In addition, a parallel analysis (Horn, 1965) was performed, which yielded a four-factor structure (Table 2) confirming the sedimentation plot (Figure 1).

**Table 2:** Principal axis factor loadings for SMBI-9 factors.

**Items**	**Factor**			
	**1**	**2**	**3**	**4**
i1 - I use the knowledge of certain experts on the Internet as a guide to medicate myself. [*Utilizo el conocimiento de ciertos expertos en internet como guía para medicarme*].	**0.691**	0.078	−0.095	−0.023
i2 - I have chosen a drug based on its packaging, brand name or advertising. [*He elegido un medicamento con base en su empaque, marca o publicidad*].	**0.644**	0.010	0.035	−0.107
i3 - I buy medicines based on recommendations from family and acquaintances. [*Compro medicamentos con base en recomendaciones de familiares y conocidos*].	**0.548**	−0.113	0.055	0.156
a1 - Doctors make us abuse the use of medications. [*Los médicos nos hacen abusar del consumo de medicamentos*].	−0.023	**0.887**	0.053	−0.097
a2 - I consider my remedies to be safer than those prescribed by a doctor. [*Considero que mis remedios son más seguros que aquellos que me receta un médico]*.	0.047	**0.482**	0.012	0.261
e1 - I avoid having to go to the doctor even if I know I have a disease. [*Evito tener que ir al médico aun sabiendo que tengo una enfermedad*].	−0.036	0.004	**0.916**	−0.090
e2 - Going to the doctor causes me anguish. [*Me causa angustia ir al médico*].	0.026	0.064	**0.436**	0.078
p1 - I take medicine in anticipation of a possible illness or disease. [*Tomo remedios anticipándome a un posible padecimiento o enfermedad*].	−0.010	−0.116	0.084	**0.709**
p2 - I decide to take medications, or natural equivalents (teas, infusions, etc.), as a way to avoid any pain or physical discomfort. [*Decido tomar medicamentos, o equivalentes naturistas (tés, infusiones, etc.), como una forma de evitar que me llegue a doler algo o tener algún malestar físico*].	−0.027	0.097	−0.103	**0.653**

*The extraction method ‘Principal Axis Factorization' was used in combination with a ‘PROMAX' rotation. Values in bold indicate the highest factor loading of the item*.

The first factor explained 13.1% of the total variance, while the second factor explained 12.2% of the total variance, the third factor explained 11.7% of the total variance and the fourth, 11.4%. The four factors explained 48.4% of the total variance. Moderate correlations were found among the factors: F1-F4 = 0.510, F1-F3 = 0.428, F1-F2 = 0.311, F2-F4 = 0.586, F2-F3 = 0.530, F3-F4 = 0.534.

Descriptive data for each factor and for the total SMBI-9 are shown in Table 3. These data correspond to the mean and standard deviation of the scores obtained by the sample.

**Table 3:** Descriptive statistics for SMBI-9 scores by gender and factor.

**Gender**	**F1**		**F2**		**F3**		**F4**		**TOTAL SMBI-9**	
	**M**	**SD**	**M**	**SD**	**M**	**SD**	**M**	**SD**	**M**	**SD**
Men	4.98	3.54	3.5	2.87	3.94	3.07	3.67	3.01	16.1	9.24
Women	5.07	3.39	3.13	2.77	3.34	3.07	3.16	2.72	14.7	8.43
Total (Both)	5.05	3.41	3.2	2.78	3.45	3.08	3.24	2.77	14.9	8.58

### 3.3 Confirmatory factor analysis

A confirmatory factor analysis was performed with the SsC sample using the maximum likelihood estimation method. The fit indices of the proposed model suggested an adequate fit χ^2^(21) = 25.616, *p* = 0.221 (χ^2^/DF = 1.220), with optimal levels CFI = 0.995, TLI = 0.991 and RMSEA = 0.022 (90% confidence interval, 0.00 Lower, 0.048 Upper. pClose = 0.963). The goodness-of-fit index, parsimony and root mean square residual also showed acceptable levels (GFI = 0.987, AGFI = 0.973, PNFI = 0.567, PCFI = 0.580, SRM*R* = 0.022).

**Figure 2:**
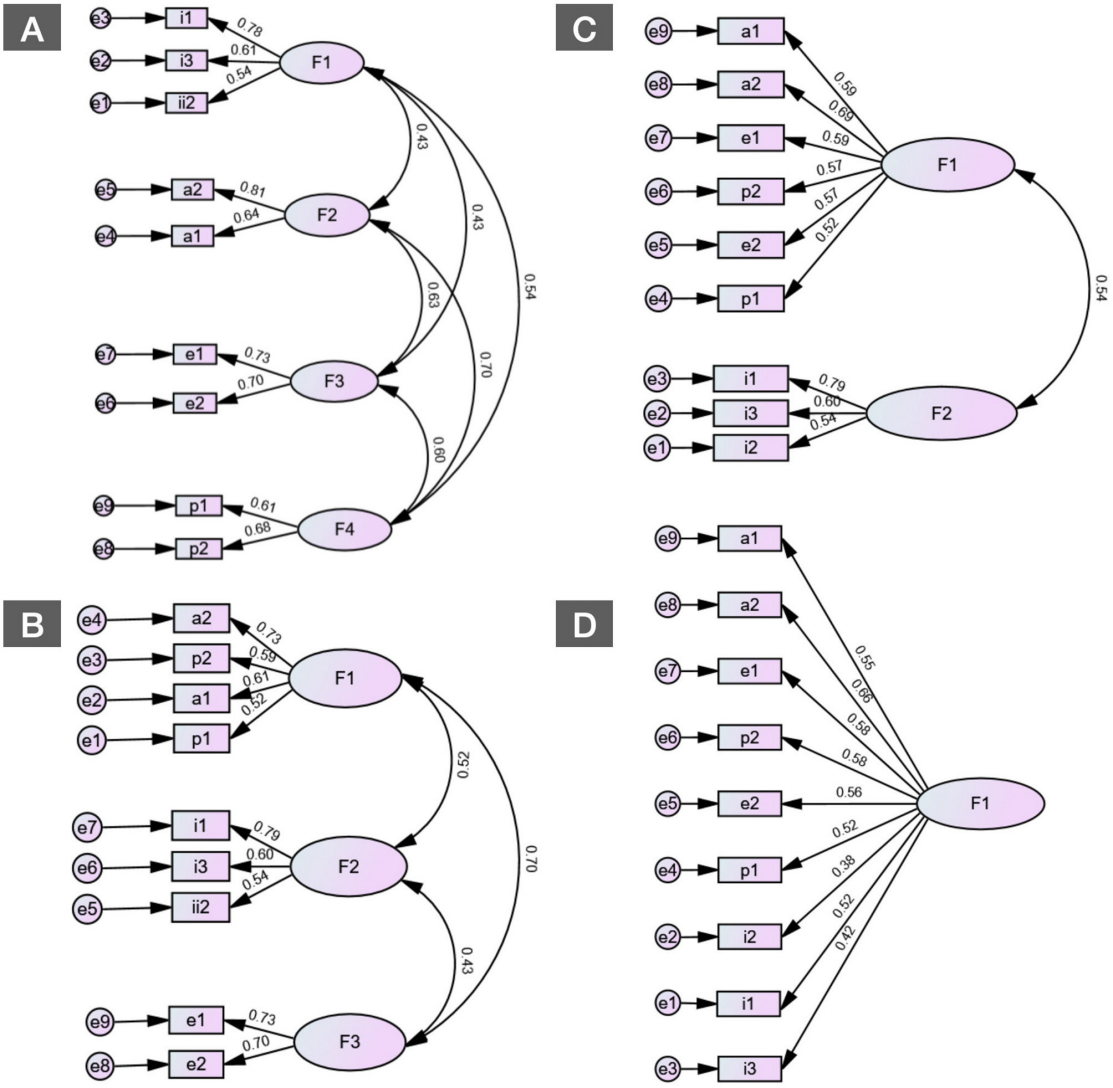
Comparative models of the Self-Medication Behavior Inventory (SMBI-9) using confirmatory factor analysis. **(A)** Four-factor model. **(B)** Three-factor model. **(C)** Two-factor model. **(D)** One-factor model.

Additionally, three other models were tested (Figure 2). The data from these models do not suggest a good fit, except for the three-factor model, but with a higher and significant chi-square value, while the four-factor model has the best overall fit, with the highest values of CFI and TLI, and the lowest RMSEA value, indicating an optimal fit. Furthermore, the Chi-square is significantly lower and not significant (Table 4).

**Table 4:** Fit indices for different CFA models of SMBI-9.

	**TLI**	**CFI**	**RMSEA**	**Chi^2^ (df)**
Unifactor model	0.695	0.771	0.129	226.431 (27), *p <* 0.001
Two-factor model	0.875	0.910	0.083	104.581 (26), *p <* 0.001
Three-factor model	0.940	0.960	0.057	58.937 (24), *p <* 0.001
Four-factor model	0.991	0.995	0.022	25.616 (21), *p =* 0.221

### 3.4 Analysis of invariance

Because the instrument was applied in two countries, the structure of the SMBI-9 was analyzed between the Colombian sample and the Mexican sample [Model unconstrained (MU)] (Table 5). The data indicated an optimal fit (CFI = 0.996, TLI = 0.993, GFI = 0.978, RMSEA = 0.015). Subsequently, this analysis was conducted on a fully constrained model (MC). The results of this also indicate an optimal fit (CFI = 0.988, TLI = 0.984, GFI = 0.97, RMSEA = 0.022). Chi-square analysis between the two models indicated no significant differences suggesting invariance (*X*^2^ MU = 45.9, df = 42, *X*^2^ MC = 61.4 df = 51, *p* > 0.05). In relation to gender, invariance was also analyzed. The data for MU indicate an acceptable fit (CFI = 0.90, TLI = 0.983, GFI = 0.976, RMSEA = 0.022), and those for MC (CFI = 0.995, TLI = 0.993, GFI = 0.974, RMSEA = 0.014). Chi-square analysis between the two models indicated no significant differences suggesting invariance (*X*^2^ MU = 51.0, df = 42, *X*^2^ MC = 51.3 df = 51, *p* > 0.05).

**Table 5:** Fit indices and chi-square results for variance analysis models.

**Index/Model**	**Country-based invariance (MU)**	**Country-based fully constrained (MC)**	**Gender-based invariance (MU)**	**Gender-based fully constrained (MC)**
CFI	0.996	0.988	0.90	0.995
TLI	0.993	0.984	0.983	0.993
GFI	0.978	0.97	0.976	0.974
RMSEA	0.015	0.022	0.022	0.014
Chi-Square (X^2^)	45.9 (df = 42)	61.4 (df = 51)	51.0 (df = 42)	51.3 (df = 51)
*p*-value^*^		>0.05		>0.05

*All abbreviations used in this table and throughout the text are described in the main body of the work and in the Abbreviations section*. ^*^Comparison between X^2^ *MU vs MC models*

### 3.5 Concurrent validity

Concurrent validity analysis was performed with the total sample (*N* = 779). The total SMBI-9 and its factors obtained moderate to strong correlations with the GSE. Correlations with the PSI-HB resulted in weak but significant correlations. SMS also resulted in a weak but significant correlation with SMBI-9, except for the influence factor (F2). Finally, the relationship between SMBI-9 and PSI-HB yielded only a weak significant correlation between PSI-HB and the influence factor (F1) of SMBI-9 (Table 6).

**Table 6:** Relationships between the SMBI-9 and other scales.

	**SMBI-9 Total**	**SMBI-9 F1 Influence**	**SMBI-9 F2 Attitude**	**SMBI-9 F3 Avoidance**	**SMBI-9 F4 Prevention**	**GSE**	**SMS**	**PSI-HB**
GSE	0.860^**^	0.616^**^	0.618^**^	0.574^**^	0.623^**^			
SMS	−0.140^**^	−0.065	−0.076^*^	−0.148^**^	−0.110^**^	−0.135^**^		
PSI-HB	0.233^**^	0.268^**^	0.087^*^	0.122^**^	0.231^**^	0.181^**^	−0.082^*^	
DAI	0.054	0.115^**^	−0.053	0.041	0.076^*^	0.087^*^	0.006	0.140^**^

*All abbreviations used in this table and throughout the text are described in the main body of the work and in the Abbreviations section*. ^**^ < *0.01*, ^*^ < *0.05*

### 3.6 Internal consistency analysis

As with the concurrent validity analysis, the internal consistency analysis also used the data from the participants of the two subsamples. Acceptable reliability was obtained (ω = 0.77). The factors yielded moderate levels of internal consistency: F1 (Influence) ω = 0.665, F2 (Attitude) ω = 0.687, F3 (Avoidance) ω = 0.665, F4 (Prevention) ω = 0.583.

### 3.7 Comparative analysis of sociodemographic variables in relation to the SMBI-9

Different analyses were performed on the total sample to compare possible differences by country, gender, and factor. A summary of these results is shown in Table 7. The total sample in this case was reduced to 774 participants because 5 of the 779 omitted some sociodemographic data and therefore were not included in these comparisons. In the ANOVA comparisons (Country × Factor) only differences were found in factor 4 “Prevention” [*F*_(1,773)_ = 4.859, *p* = 0.028, = 0.006], with higher scores in Mexico ($\bar{X}$ Colombia = 3.09, $\bar{X}$ Mexico = 3.56). In the comparisons for Gender x Factor, differences were found only in F3 “Avoidance” [*F*_(1,773)_ = 4.287, *p* = 0.039, = 0.006], with a higher mean in males ($\bar{X}$ Males = 3.94, $\bar{X}$ Females 3.34).

**Table 7:** Summary of findings of the comparative analyses of SMBI-9 and other sample variables.

**Statistical test**	**Variables**	**Factor influence**	**Factor attitude**	**Factor avoidance**	**Factor prevention**
ANOVA Two-way	Country × Factor	>0.05	>0.05	>0.05	**<** **0.05**
	Gender × Factor	>0.05	>0.05	**<** **0.05**	>0.05
ANOVA One-way	Schooling	**<** **0.05**	>0.05	>0.05	>0.05
	Health insurance	>0.05	>0.05	**<** **0.05**	>0.05
t	Health condition	>0.05	>0.05	**<** **0.05**	>0.05
	Knowledge of medicine	**<** **0.05**	**<** **0.05**	**<** **0.001**	**<** **0.001**
r	Income Level	***r** **=*** **081**, ***p** **=*** **0.035**	>0.05	>0.05	>0.05
	Age	***r** **=*** **- 0.140**, ***p** **=*** **<** **0.001**	***r** **=*** **0.081**, ***p** **=*** **0.024**	***r** **=*** **−0.073**, ***p** **=*** **0.041**	>0.05

Values in bold indicate statistically significant differences or correlations.

Factors were compared by level of schooling using one-way ANOVA. Differences were only found in F1 “Influence” [*F*_(4,773)_ = 33.384, *p* = 0.021]. *Post-hoc* comparisons with Games–Howell yielded significant differences in the comparisons at the Elementary vs. High School (*p* = 0.003), Elementary vs. Undergraduate (*p* = 0.003) and Elementary vs. Graduate (*p* = 0.022) levels of education.

Differences were also found in relation to having health insurance, specifically in F3 “Avoidance” [*F*_(2,773)_ = 3.421, *p* = 0.033]. *Post-hoc* analyses indicate that the differences are between the Uninsured vs. Public Insurance (*p* = 0.027) and Uninsured vs Private Insurance (*p* = 0.046) groups. No differences were found between having public and private insurance. The mean for the uninsured group at F3 = 4.41, while for public insurance = 3.35, and for private insurance = 3.36.

In the comparisons of people with illnesses (at least 6 months with the illness), differences were found only in F3 [*t*_(772)_ = 2.139, *p* = 0.033, *d* = 0.179], with higher scores in people with ailments.

One of the items on sample characteristics asked whether one had formal knowledge of medicine. Comparisons were made based on these two categories. In all cases, the category “without formal knowledge of medicine” obtained higher scores than the condition “having formal knowledge” and differences were found in factors F1 [*t*_(772)_ = 2.07, *p* = 0.039, *d* = 0.28], F2 [*t*_(772)_ = 2.132, *p* = 0.033, *d* = 0.305], F3 [*t*_(772)_ = 3.222, *p* = 0.001, *d* = 0.451] and in the SMBI-9 total [*t*_(772)_ = 3.241, *p* = 0.001, *d* = 0.431].

Income level had no relationship with the scale, except with F1 but with a weak relationship (*r* = −0.81, *p* = 0.035). The sample for this analysis was smaller (*N* = 673), as some participants answered “I don't know” and others “I know but I prefer not to answer.” Age showed a weak relationship with F1 “Influence” (*r* = −0.140, *p* = < 0.001), F2 “Attitude” (*r* = 0.081, *p* = 0.024), and F3 “Avoidance” (*r* = −0.073, *p* = 0.041).

## 4 Discussion

The data from this study indicate that the SMBI-9 possesses adequate psychometric properties to investigate variables related to self-medication behavior. According to our findings, factors such as avoidance, prevention, attitudes toward physicians, and social influence significantly impact self-medication behavior and constitute an optimal model according to confirmatory factor analysis.

Influence (F1) of sources from family members, commercial advertisements, Internet sources, according to our results, plays an important role in self-medication behavior. This suggests that professional information coming from physicians or health specialists is not considered when self-medicating. Indeed, it has been reported that people seeking health information give credibility to a website based on its design and show little concern for the credibility of the sources or the identities of the contributors behind the information on the site (Eysenbach, 2002).

The Attitude factor (F2) supports the findings of other studies indicating that that public trust in medicine is affected by their attitudes, and this trust has decreased due to the influence of the media, which focus attention on uncertainty, medical errors and conflicts of interest (Mechanic, 1996). Furthermore, the spread of false health-related news not only increases self-medication but also undermines the professional-patient relationship (Barreto et al., 2021), and fosters mistrust toward medicines (Pound et al., 2005).

The Prevention factor (F4) is an anticipatory behavior in relation to undesirable symptoms or the threat of a condition. This supports other findings indicating a relationship between self-medication and fear of illness during the COVID-19 pandemic (Faraji et al., 2022). Furthermore, some individuals may view self-medication, including vitamins and supplements, as a proactive way to stay healthy, preventing potential health problems (Santos and Barros Filho, 2002; Lawand et al., 2023). This could happen even for chronic conditions or recurrent diseases (Lin et al., 2022).

The confirmation of the Avoidance factor (F3) in self-medication behavior, in addition to what has already been pointed out in the introduction, could reflect that individuals who avoid medical care may be more inclined to resort to self-medication because they are afraid of what a doctor might tell them. People with higher levels of anxiety are more prone to avoid medical care (Ganson et al., 2020). Self-medication can be seen as a way to take control of one's own health and avoid feeling powerless in the face of illness. It is likely that the relationship found between this factor and GSE reinforces the above. In this regard, there is evidence of a relationship between perceived control and self-efficacy (Salehi et al., 2016; Li et al., 2018).

The relationships found between SMBI-9 and GSE, which confirm our hypotheses, is one of the most relevant findings. This implies that people with high self-efficacy feel more capable of managing their health problems without the need to consult a physician. In this sense, it has been suggested that confidence in self-care reflects self-efficacy (Vaughan Dickson et al., 2017). Indeed, self-medication is a form of self-care (Lifshitz et al., 2020). But in addition to self-care and the benefits related to the usefulness of self-medication to treat minor health problems, there may also be important risks (Hughes et al., 2001). The level of self-efficacy could influence how people assess these risks and benefits. For example, it has been noted that people with high self-efficacy may engage in riskier behaviors (Krueger and Dickson, 1994).

Similar to self-efficacy, a positive relationship was found between the SMBI-9 and social influence. This is consistent with indications that advertising (Burak and Damico, 2000; Fuentes Albarrán and Villa Zapata, 2008), social networks (Zeb et al., 2022), and close individuals (Anghel and Craciun, 2013), influence self-medication behavior. Such findings are also consistent with the theory of reasoned action, which emphasizes the impact of normative beliefs, i.e., what significant others expect an individual to do (Hagger et al., 2002).

The weak but significant relationships between the SMS and the SMBI-9 can be interpreted as supporting the evidence for the validity of SMBI-9. As hypothesized, the relationship between the SMS and the SMBI-9 was negative. This is because most of the items of the SMS have a direction contrary to self-medication, For instance, items like if I am in pain “I try to ignore it and move on” or “I prefer to let my body fight.” Items that have a positive direction were reverse scored to maintain consistency with the overall SMS score. Notably variables such as health literacy, educational level and age that have been reported to be related to SMS (Alqarni et al., 2023), also had weak but significant relationships with SMBI-9. This may also be an indicator of evidence of validity.

The DAI, as observed in the results, had only a weak but significant relationship with F1 of the SMBI-9. Although relationships with all factors were expected, it is likely that attitudes toward medication as measured by the DAI are not related to self-medication. For example, it has been reported that samples of pharmacobophobic vs. pharmacophilic individuals, as a function of their scores with the DAI, show no differences in self-medication behavior (Lucca et al., 2022).

The differences observed between people with formal health training and, therefore, higher health literacy, and those without such training, support findings from previous studies (Amiri et al., 2022; Alqarni et al., 2023). These differences underscore the importance of promoting health literacy as a preventive measure against the risks associated with self-medication. Actions or initiatives in this direction have been undertaken, for example, by the Observatory of Self-medication Behavior [*Obervatorio del Comportamiento de la Automedicación* (OCAM)] (Calderón et al., 2020).

### 4.1 Limitations

This is the first version of the SMBI-9, and therefore there are psychometric properties that require improvement, for example, the reliability coefficients of the instrument. These coefficients show a moderate level and can therefore be considered satisfactory (Vera et al., 2014), in addition to the fact that they are derived from the first finished version of the test (Nunnally and Bernstein, 2010). To this should be added that the SMBI-9 has few items (Jackson and Verberg, 2006). Even so, it is important to increase the coefficients. It is likely that the inclusion of more items in other versions of the instrument would help to increase the reliability coefficients.

The SMBI-9 is focused on assessing variables linked to self-medication behavior and which, according to the literature and our findings, contribute to explaining it. However, it does not explicitly assess the intensity or frequency of such behavior. Future versions of the instrument could include a related factor, or future studies could address this relationship.

Another limitation of the study is that participants were recruited on the basis of their response to an invitation, so it is desirable that future studies select participants by random sampling.

The instrument showed invariance in two nations, with cultural similarities and different regulations on access policies to drugs that are not usually sold without a prescription. Even so, it is worth reviewing whether the findings can be generalized to other populations with greater or lesser regulation and greater or lesser access to health services.

It is recommended that future studies conduct sensitivity analyses to examine how variations in the test items, participant responses, and analysis methods can affect crucial metrics such as the reliability and validity of the instrument. In this study, although a specific sensitivity analysis was not performed, the invariance and confirmatory factor analyses provided valuable insights into the stability and robustness of the test under different conditions and models. These analyses suggest a certain robustness of the instrument, but we also highlight the need for future research to further explore these issues, including the addition of more items per factor and the exploration of incremental validity, among other sensitivity aspects.”

### 4.2 Conclusions

The principal contribution of this study on self-medication behavior is that it is the first instrument that evaluates, in a Mexican and Colombian sample, variables that explain such behavior instead of limiting itself to measuring its intensity and frequency. In addition, it has adequate psychometric properties and probably requires little adaptation to be applicable to other samples or populations.

The SMBI-9 encompasses four factors (influence, attitude, avoidance, prevention) and only 9 items, swift administration and easy integration with other measures in future research.

The extracted factors suggest a potential sequential or concurrent relationship among them. For instance, negative attitudes toward doctors and medicine could lead an individual to avoid the conventional medical system and, instead, become more dependent on social influences as sources of information for self-medication. It would be particularly relevant to address these relationships in upcoming studies. In the future, with more data, statistical treatments can be developed to understand and compare different models of self-medication behavior.

Our findings establish a foundation for the creation of interventions and educational programs. These initiatives can link to the factors outlined in the SMBI-9 model and assist individuals in securing accurate information and support, thereby empowering informed decision-making about self-medication.

## Data availability statement

The raw data supporting the conclusions of this article will be made available by the authors, without undue reservation.

## Ethics statement

The studies involving humans were approved by the Ethics Committee of Universidad de las Américas Puebla. The studies were conducted in accordance with the local legislation and institutional requirements. The participants provided their written informed consent to participate in this study.

## Author contributions

JP-C: Conceptualization, Data curation, Formal analysis, Investigation, Methodology, Project administration, Resources, Supervision, Validation, Writing—original draft, Writing—review & editing. MO-B: Investigation, Methodology, Resources, Validation, Writing—review & editing. RH-R: Conceptualization, Data curation, Investigation, Methodology, Resources, Validation, Writing—review & editing. YO-R: Conceptualization, Investigation, Methodology, Resources, Validation, Writing—review & editing. RG: Conceptualization, Investigation, Methodology, Resources, Validation, Writing—original draft. AP-A: Conceptualization, Investigation, Methodology, Resources, Writing—review & editing.
